# Characterization of H5N1 high pathogenicity avian influenza virus belonging to clade 2.3.4.4b isolated from Ezo red fox in Japan in a mouse model

**DOI:** 10.1128/spectrum.01097-25

**Published:** 2025-11-26

**Authors:** Shintaro Shichinohe, Takahiro Hiono, Yasushi Itoh, Kosuke Takada, Yurie Kida, Pei Wang, Daisuke Motooka, Norikazu Isoda, Ayato Takada, Yoshihiro Sakoda, Tokiko Watanabe

**Affiliations:** 1 Department of Molecular Virology, Research Institute for Microbial Diseases, The University of Osaka 13013, Suita, Osaka, Japan; 2 Laboratory of Microbiology, Faculty of Veterinary Medicine, Hokkaido University 12810, Sapporo, Hokkaido, Japan; 3 International Collaboration Unit, International Institute for Zoonosis Control, Hokkaido University 12810, Sapporo, Hokkaido, Japan; 4 One Health Research Center, Hokkaido University 12810, Sapporo, Hokkaido, Japan; 5 Division of Pathogenesis and Disease Regulation, Department of Pathology, Shiga University of Medical Science 13051, Otsu, Shiga, Japan; 6 Genome Information Research Center, Research Institute for Microbial Diseases, The University of Osaka 320555 , Suita, Osaka, Japan; 7 Division of Global Epidemiology, International Institute for Zoonosis Control, Hokkaido University 12810, Sapporo, Hokkaido, Japan; 8 Hokkaido University Institute for Vaccine Research and Development (HU-IVReD), Hokkaido University 12810, Sapporo, Hokkaido, Japan; 9 Center for Infectious Disease Education and Research (CiDER), The University of Osaka 13013, Suita, Osaka, Japan; 10 Center for Advanced Modalities and DDS (CAMaD), The University of Osaka 13013, Suita, Osaka, Japan; Universiteit Utrecht, Utrecht, the Netherlands

**Keywords:** H5N1 high pathogenicity avian influenza virus, pathogenicity, mouse model

## Abstract

H5N1 high pathogenicity avian influenza virus (HPAIV) has spread in wild birds and poultry worldwide. H5N1 HPAIV belonging to the currently predominant clade 2.3.4.4b has infected not only birds but also mammals (wild and domestic animals), with several human infections also being reported, raising concerns for public health. In 2022, a clade 2.3.4.4b H5N1 HPAIV strain, A/Ezo red fox/Hokkaido/1/2022 (H5N1; Fox/Hok/1/22), was isolated from an Ezo red fox (*Vulpes vulpes schrencki*) in Hokkaido, Japan; this was the first reported case of clade 2.3.4.4b H5N1 HPAIV isolation from a mammalian species in Japan. Several amino acid substitutions in the PB2 protein play an important role in the adaptation of avian influenza viruses to mammals, but Fox/Hok/1/22 PB2 does not have any of these well-known mammalian-adapting PB2 substitutions. Here, we investigated the biological properties of Fox/Hok/1/22 in a mouse model and found that this virus was highly virulent in mice and replicated well in multiple organs, including the lungs and brain. We then examined whether viruses isolated from these organs acquired known mammalian-adapting PB2 amino acid substitutions, such as PB2 E627K. Deep sequencing analysis of viral RNA from mouse brain and lungs revealed that virus with PB2-627E was predominant in three of four mice, whereas the PB2-627K substitution was predominant in one mouse. These results indicate that Fox/Hok/1/22 is highly virulent in mice despite lacking known PB2 substitutions involved in mammalian adaptation.

**IMPORTANCE:**

The H5N1 avian influenza virus has caused severe disease in birds worldwide and is now spreading to mammals, including humans. In 2022, this virus was detected for the first time in an Ezo red fox in Japan. To understand its potential impact on mammals, we studied this virus in mice and found that it caused severe illness, spreading to multiple organs, including the lungs and brain. Surprisingly, despite lacking genetic mutations typically associated with mammalian adaptation, the virus was highly virulent in mice. This finding suggests that the H5N1 virus may pose a greater threat to mammals, including humans, than previously thought. Given their continued spread among wild and domestic animals, our findings underscore the urgent need to monitor how recent H5N1 viruses behave in mammals.

## INTRODUCTION

Since the emergence of H5N1 high pathogenicity avian influenza virus (HPAIV) of the A/goose/Guangdong/1/1996 (Gs/GD) lineage, H5N1 HPAIVs have spread due to wild bird migration across many regions of the world. As of late 2021, H5N1 HPAIVs belonging to clade 2.3.4.4b have spread globally, replacing H5N8 HPAIVs as the main epidemic subtype (1). The spread of clade 2.3.4.4b H5N1 HPAIV has been unprecedented, reaching not only Asia, Europe, North America, Oceania, and Africa, but also South America and Antarctica, where there had previously been no or few outbreaks of H5N1 HPAIV.

Clade 2.3.4.4b H5N1 HPAIVs have infected a variety of mammalian species in the order Carnivora, including red foxes and skunks (2). In 2022, minks on farms in Spain and Finland were infected with clade 2.3.4.4b H5N1 HPAIV, causing mass mortality (3, 4). Infections in cats have been reported worldwide, presumably due to raw and inadequate heating of poultry meat or milk from virus-infected cattle (5–7). Marine mammals, such as seals and dolphins, have also been infected, with mass deaths reported in North and South America (8). More recently, an outbreak of H5N1 HPAIV in cattle has raised serious concerns in the United States, as infections of farm workers were also reported (9). Between April 2022 and 20 March 2025, 70 confirmed human cases of infection with clade 2.3.4.4b H5N1 HPAIV were reported in the United States (9), raising concerns about a pandemic threat. Therefore, to assess the risk of future spread of these clade 2.3.4.4b viruses to other mammalian species and humans, it is important to characterize the properties of these viruses in mammalian models.

In March 2022, a clade 2.3.4.4b H5N1 HPAIV, A/Ezo red fox/Hokkaido/1/2022 (H5N1; Fox/Hok/1/22), was isolated from a dead Ezo red fox (*Vulpes vulpes schrencki*) at a public garden in Sapporo in Hokkaido, Japan (10). Viral meningoencephalitis and moderate viral replication in the upper respiratory tract were observed in the dead fox; however, the biological properties of this virus in mammals have not been well-studied. Therefore, here, we characterized the biological features of Fox/Hok/1/22 in a mouse model.

## MATERIALS AND METHODS

### Cells and viruses

Madin-Darby canine kidney (MDCK) cells were kindly provided by Prof. Yoshihiro Kawaoka. MDCK cells were cultured in Eagle’s minimal essential medium (MEM) containing 5% newborn calf serum. A549 (human lung epithelial) cells were obtained from American Type Culture Collection (ATCC) and maintained in Ham’s F-12K medium (Wako, Osaka, Japan) containing 10% fetal calf serum (FCS) at 37°C in 5% CO_2_. DF-1 cells were obtained from ATCC and maintained in DMEM medium (Nacalai tesque, Kyoto, Japan) containing 10% FCS at 39°C in 5% CO_2_. Fox/Hok/1/22 (H5N1) (10) was isolated in 10-day-old chicken eggs from dead fox brain homogenate and then propagated for mouse infection and titrated by use of plaque assays on MDCK cells cultured in Eagle’s MEM containing 0.3% BSA and 1 µg/mL *N-p*-tosyl-L-phenylalanin chloromethyl ketone (TPCK)-treated trypsin. Virus stocks were stored at –80°C until use. All viral experiments were performed under biosafety level 3 conditions and approved by the Institutional Review Board of the Research Institute for Microbial Diseases, The University of Osaka (protocol number: BIKEN-00311-005) and the Animal Research Committee of the Research Institute for Microbial Diseases, The University of Osaka (approval number, R04-04-0), including approval for potential emergence of mammalian-adapting mutations.

### Virus growth kinetics in cell culture

A549 and DF-1 cells were infected in duplicate with virus at a multiplicity of infection of 0.01. The culture medium was removed, and cells were washed once with PBS. The virus was inoculated and incubated at 37°C for A549 cells with Ham’s F-12K medium containing 0.3% bovine serum albumin and 0.5 µg/mL TPCK-trypsin. The virus was inoculated and incubated at 39°C for DF-1 cells with 1× DMEM containing 0.3% bovine serum albumin and 0.5 µg/mL TPCK-trypsin. Samples were collected at 0, 6, 12, 24, 48, and 72 h post-infection. Virus titers at the indicated time points were determined by performing plaque assays in MDCK cells. Data are the mean  ±  s.d. of three independent experiments.

### Animal experiments

Five-week-old female BALB/c mice were obtained from Japan SLC (Shizuoka, Japan). To determine the 50% mouse lethal dose (MLD_50_), the mice were intranasally inoculated with 0.1, 1, 10, 10^2^, or 10^3^ plaque-forming units (PFU) (in 50 µL) of Fox/Hok/1/22 under isoflurane anesthesia. Body weight change and survival were monitored daily for 14 days. Virus-infected mice were euthanized if they lost more than 25% of their initial body weight. MLD_50_ values were calculated according to the method of Reed and Muench. Virus titers in various organs were determined by use of plaque assays on MDCK cells. To examine virus titers, four mice per group were intranasally infected with Fox/Hok/1/22 at 10^3^ PFU. At 3 and 6 days post-infection (dpi), the animals were euthanized, and brain, nasal turbinate, lungs, heart, spleen, liver, kidney, and colon were collected. The virus titers in organs were determined by performing plaque assays on MDCK cells. All animal experiments were approved by the Animal Research Committee of the Research Institute for Microbial Diseases, The University of Osaka (approval number, R04-04-0).

### Histological examination

On day 6 after virus infection, brain and lung tissue samples were fixed with 10% formalin and embedded in paraffin. Sections were stained with hematoxylin and eosin. Influenza virus nucleoprotein (NP) antigens were stained with antiserum from rabbits immunized with an NP synthetic peptide (AFTGNTEGRTSDMR at positions 428–441 of the NP sequence; GenBank accession number, ADC34563) after heat antigen retrieval for 15 min in 0.01 M citrate-phosphate buffer. After incubation with anti-rabbit immunoglobulin antibody conjugated with horseradish peroxidase (Nichirei Bioscience Inc., Tokyo, Japan), NP was detected with diaminobenzidine (Nichirei Biosciences Inc., Tokyo, Japan).

### Sequencing analysis

Viral RNA was extracted from homogenates of brain and lungs by using the QIAamp Viral RNA Mini kit (Qiagen, Hilden, Germany) according to the manufacturer’s instructions. Universal primer sets for influenza A virus were used for RT-PCR of all eight gene segments (11). PCR products were ligated with gene-specific primers and sequenced by Eurofins Genomics K.K. (Tokyo, Japan). Sequencing data were analyzed using GENETYX version 22.0.1 (Genetyx Corporation, Tokyo, Japan).

For deep sequencing analysis of mutant viruses, library preparation was performed using a KAPA RNA hyperprep kit (Kapa Biosystems, MA, USA) or a TruSeq stranded Total RNA Library Prep. Kit with RiboZero plus (Illumina, CA, USA) according to the manufacturer’s instructions. Sequencing was performed on a MiSeq (Illumina) or NovaSeq 6000 sequencer (Illumina) in the 151-base paired-read mode. The raw FASTQ format data files obtained from the sequencing samples were filtered, and adaptors and low-quality reads were removed by using CLC Genomics Workbench software (CLC bio, Aarhus, Denmark). Filtered reads were mapped to the reference Fox/Hok/1/22 (from EPI2021929 to EPI2021936). From the mapped reads, more than 0.1% of mutations were extracted. The percentage of mutants was calculated as PB2-627E if the nucleotide of PB2 at 1892 in the full-length genome sequence was guanine and as PB2-627K if it was adenine.

## RESULTS

### Replicative ability of Fox/Hok/1/22 *in vitro* and *in vivo*

Fox/Hok/1/22 was previously isolated from a dead Ezo red fox in Japan (10). Necropsy of the dead fox showed viral meningoencephalitis in the brain with high virus titers and moderate viral replication in the respiratory tract (10). We found that Fox/Hok/1/22 possesses several amino acid substitutions in its viral proteins that are known to be associated with the adaptation of avian influenza viruses to mammalian hosts (i.e., five amino acids in PB2, two in PB1, three in PA, three in HA, one in NP, three in M, and five in NS1 as shown in Table S1); however, this virus does not possess well-known mammalian-adapting substitutions in PB2, such as E627K or D701N, both of which are strongly associated with increased virulence of avian influenza viruses in mammalian hosts (12, 13).

**Fig 1:**
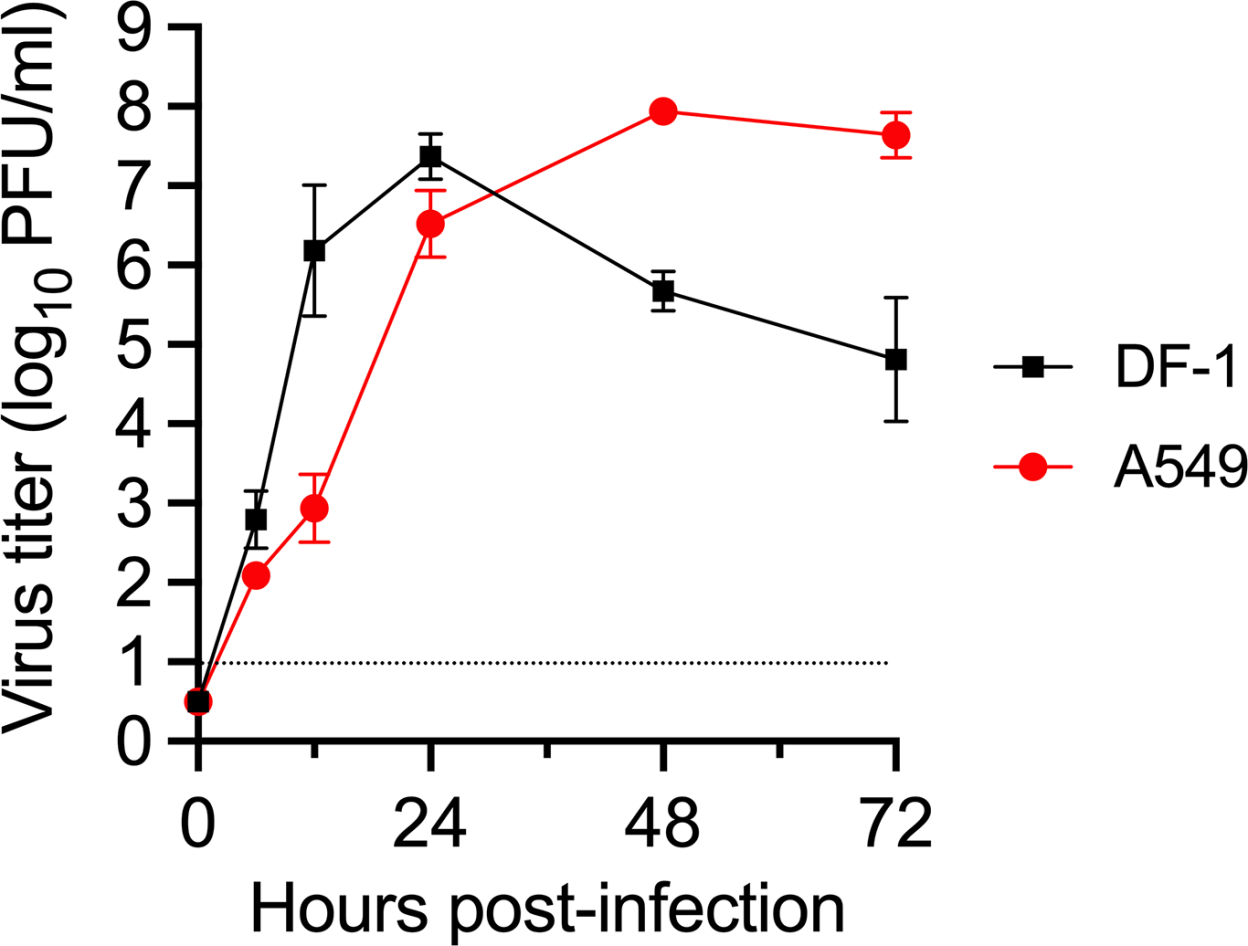
Replicative ability in human lung- and chicken-derived cells. Growth kinetics of Fox/Hok/1/22 in A549 and DF-1 cells. Human lung-derived A549 and chicken-derived DF-1 cells were infected with Fox/Hok/1/22 at a multiplicity of infection of 0.01. Culture supernatants were collected at 0, 6, 12, 24, 48, and 72 hpi, and virus titers were determined by performing plaque assays on MDCK cells. Data are the mean  ±  s.d. of three independent experiments. The detection limit (1.0 log₁₀ PFU/g) is indicated by the dotted line.

To investigate the replicative ability of Fox/Hok/1/22 *in vitro*, we examined its growth kinetics in chicken embryo fibroblast DF-1 cells and human lung A549 cells. Fox/Hok/1/22 grew well in DF-1 cells at 39°C with a peak titer of 7.37 ± 0.29 log_10_ PFU/mL at 24 h post-infection (hpi) (Fig. 1). In A549 cells, Fox/Hok/1/22 replicated efficiently with a peak titer of 7.94 ± 0.15 log_10_ PFU/mL at 48 hpi (Fig. 1). This result indicates that Fox/Hok/1/22 replicates efficiently in both avian DF-1 cells and human lung A549 cells.

Next, we examine the replicative ability of the virus *in vivo*. Four BALB/c mice were infected intranasally with 10^3^ PFU of Fox/Hok/1/22, and multiple organs, including brain, spleen, liver, kidney, colon, heart, nasal turbinate, and lungs, were collected from the infected mice at 3 and 6 dpi to determine virus titers. Body weight was also monitored in this group, and the mice showed body weight loss by 6 dpi (data not shown). The mean virus titers in the lower respiratory tract (i.e., lungs) at 3 and 6 dpi were 7.43 ± 0.23 log_10_ PFU/g and 7.21 ± 0.53 log_10_ PFU/g, respectively (Table 1). In the upper respiratory tract (i.e., nasal turbinate), the mean virus titer of the virus-infected mice was 5.95 ± 1.51 log_10_ PFU/g at 6 dpi. Fox/Hok/1/22 replicated systemically in the infected mice, and the virus was recovered from multiple organs (i.e., brain, spleen, liver, kidney, colon, and heart) collected from the infected mice (Table 1). In the brains, 5.18 and 2.42 log_10_ PFU/g of virus were recovered from 2 of 4 mice at 3 dpi, and virus was recovered from all four infected mice at 6 dpi (mean virus titer = 6.79 ± 1.52 log_10_ PFU/g). These results indicate that Fox/Hok/1/22 replicates efficiently not only in the respiratory tract (i.e., lungs and nasal turbinate) of infected mice, but also causes systemic infection.

**TABLE 1:** Virus titers in organs of Fox/Hok/1/22-infected mice^*a*^

Days post-infection (dpi)	Mouse no.	Virus titer (log_10_ PFU/g)							
		Brain	Spleen	Liver	Kidney	Colon	Heart	Nasal	Lung
3	1	5.18	7.95	5.16	5.88	4.79	6.16	3.24	7.40
	2	–^*b*^	4.35	2.22	2.22	–	3.51	–	7.11
	3	–	4.94	–	–	–	2.46	–	7.62
	4	2.42	4.80	2.26	–	–	4.31	–	7.58
6	5	7.48	3.38	2.70	5.63	3.37	6.51	6.14	6.79
	6^*c*^	8.32	6.65	6.79	6.39	4.09	7.91	7.86	7.99
	7	6.59	2.57	–	2.79	–	4.97	5.57	7.11
	8	4.78	–	2.10	3.34	–	6.35	4.22	6.96

^*a*^ BALB/c mice were inoculated intranasally with 10^3^ PFU (in 50 µL) of virus. Four mice were euthanized on days 3 and 6 post-infection. Multiple organs were collected for virus titration in plaque assays.

^*b*^ “–” indicates virus was not detected (detection limit: 1.8 log_10 _PFU/g).

^*c*^ Since mouse no. 6 died at 5 dpi, organs were collected at 5 dpi.

### Pathogenicity of Fox/Hok/1/22 in mice

**Fig 2:**
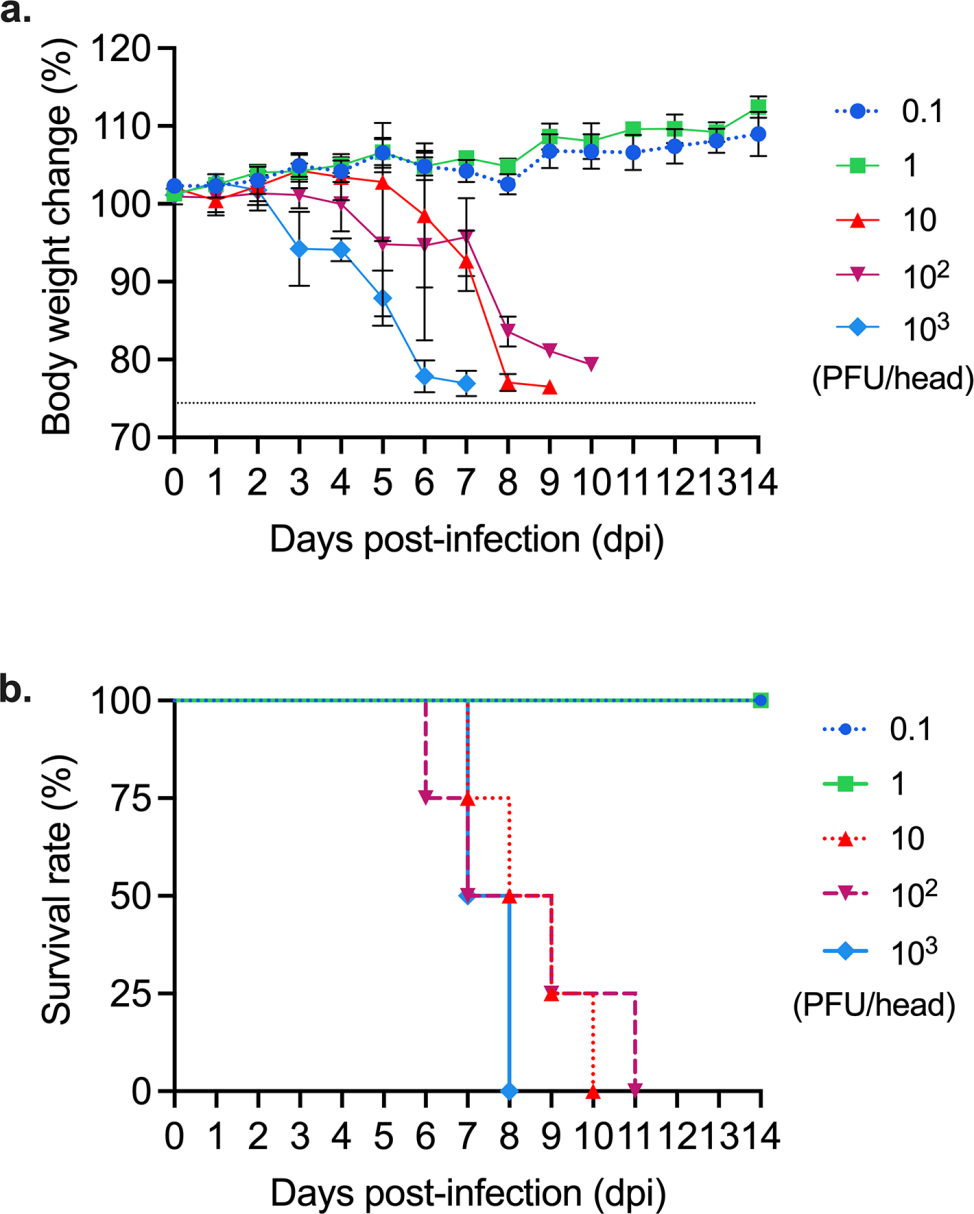
Pathogenicity of Fox/Hok/1/22 in mice. Mice were infected with 0.1 to 10^3^ PFU (in 50 µL) of Fox/Hok/1/22. (**a**) Body weight changes were monitored daily from −2 to 14 dpi. The average weight of the four mice in each group from −2 to 0 dpi was calculated as 100%. Body weight changes are shown as the mean ± s.d. of four mice per group. (**b**) Survival was calculated as death if the animal died or lost more than 25% of its initial body weight and had to be euthanized.

To examine the pathogenicity of Fox/Hok/1/22, four BALB/c mice per group were inoculated intranasally with 0.1, 1, 10, 10^2^, or 10^3^ PFU of virus and body weight changes and survival of the infected animals were monitored for 2 weeks (Fig. 2a). All mice inoculated with more than 10 PFU of Fox/Hok/1/22 exhibited severe weight loss and died by 11 dpi, whereas all mice inoculated with less than 1 PFU of virus survived (Fig. 2a and b). The 1 MLD_50_ value (amount of virus required to kill 50% of infected mice) of Fox/Hok/1/22 was 10^0.5^ PFU. In addition, some mice infected with Fox/Hok/1/22 showed neurological symptoms, such as convulsions (data not shown). These results indicate that Fox/Hok/1/22 is highly virulent in mice.

**Fig 3:**
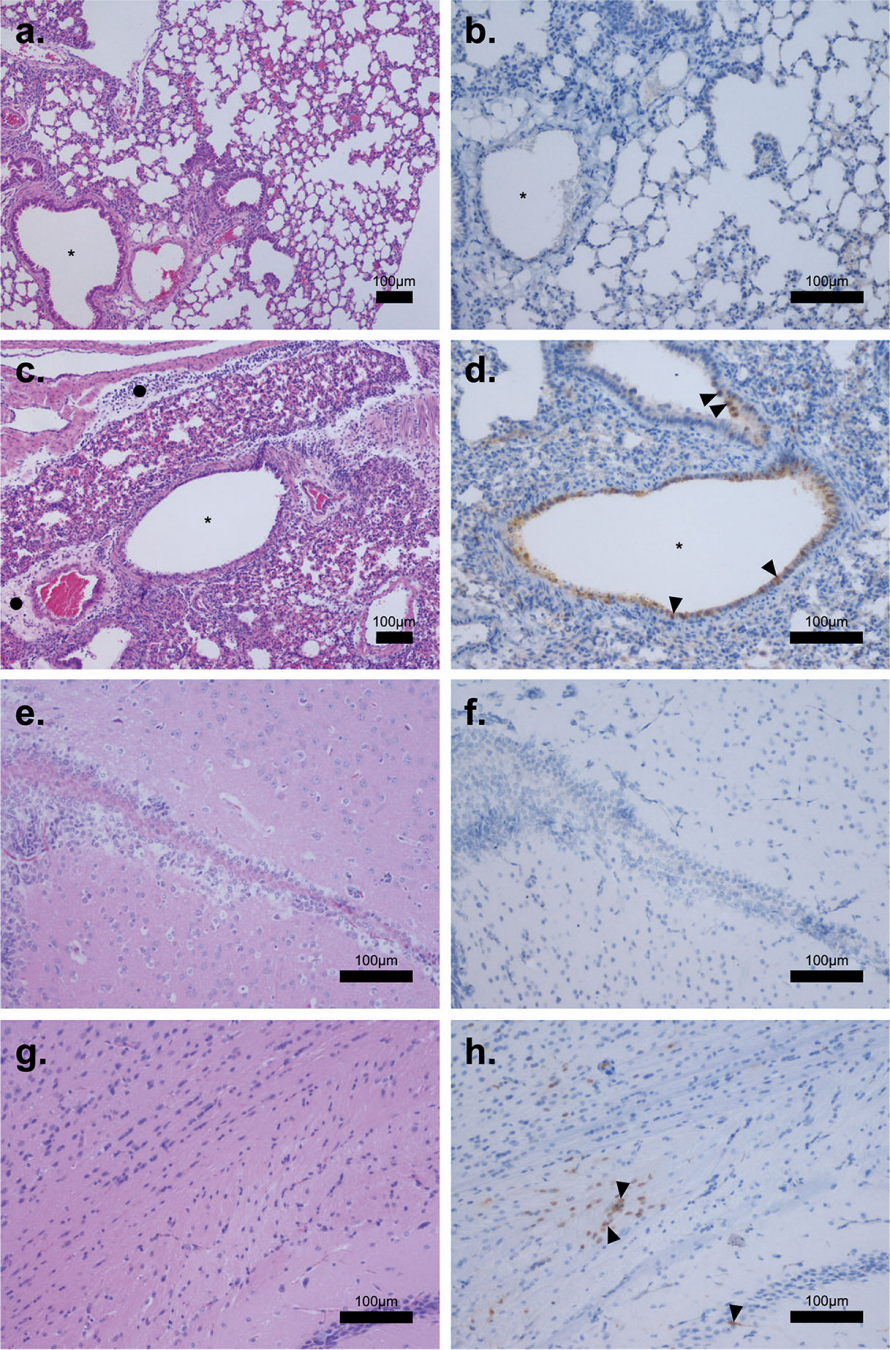
Histological analysis and distribution of viral antigens in the lungs and brain of mice infected with Fox/Hok/1/22. Lungs (**a–d**) and brain (**e–h**) were collected from mice on day 6 after inoculation with PBS (**a, b, e, and f**) or Fox/Hok/1/22 (**c, d, g, and h**). Tissues stained with hematoxylin and eosin (**a, c, e, and g**) (bar: 100 µm) or immunohistochemistry for influenza NP antigen, shown by the black arrowhead (**b, d, f, and h**) (bar: 100 µm). The asterisks indicate bronchioles.

Next, we histopathologically examined the lungs and brain of the mice infected with 10^3^ PFU at 6 dpi (Fig. 3). Alveolar spaces were smaller in the infected mice than in the uninfected mice due to swelling of alveolar epithelial cells. Lymphocytes had infiltrated the perivascular edematous space around the bronchioles of the Fox/Hok/1/22-infected mice (Fig. 3c, black circles), but not in the uninfected mice (Fig. 3a). Viral antigen (i.e., influenza A virus nucleoprotein) was detected in the bronchial epithelial cells of the Fox/Hok/1/22-infected mice (Fig. 3d), but not in uninfected mice (Fig. 3b), suggesting that Fox/Hok/1/22 caused viral pneumonia in the lungs. In contrast, in the brain, no pathological changes in cell morphology were observed in the virus-infected mice (Fig. 3e and g), although viral antigen was detected in their glial cells (Fig. 3f and h).

### Variants in the virus population isolated from the lungs and brains of Fox/Hok/1/22-infected mice

To examine whether viruses with amino acid substitutions that are known to be associated with adaptation of avian influenza viruses to mammalian hosts emerged in the mice infected with Fox/Hok/1/22, we sequenced the full-length viral genomes isolated directly from the lungs and brains of the infected mice (nos. 5, 6, 7, 8) at 6 dpi using Sanger sequencing. Two amino acid substitutions that differed from the sequence of the inoculated virus were found at positions S489P and E627K of PB2 in viruses isolated from the brain and lung of animals 5 and 6, respectively (Table 2).

**TABLE 2:** Amino acid sequences of viruses in organs of mice before and after Fox/Hok/1/22 infection by Sanger sequencing^*a*^

Gene	Amino acid^*b*^	Mouse #5		Mouse #6		Mouse #7		Mouse #8	
		Lung	Brain	Lung	Brain	Lung	Brain	Lung	Brain
PB2	489	S→P	S→P	–^*c*^	–	–	–	–	–
	627	–	–	E→K	E→K	–	–	–	–

^*a*^ Viral RNA was extracted directly from the organs of mice infected with Fox/Hok/1/22. The first-strand cDNA was synthesized by using Uni12 primer. After amplification, the PCR products were sequenced and analyzed.

^*b*^ The numbering starts with the initial methionine (position 1).

^*c*^ No mutation was detected.

**Fig 4:**
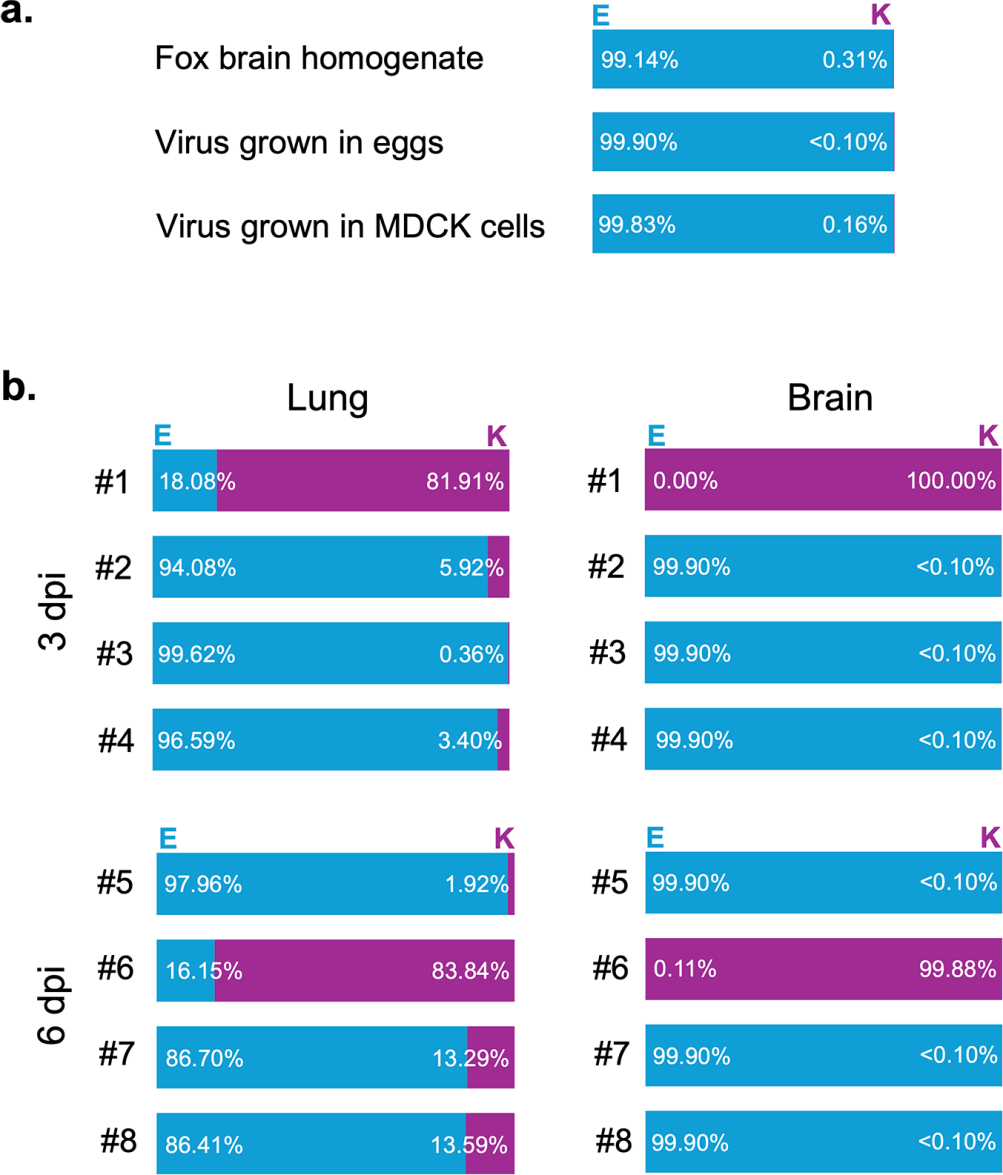
Emergence of the PB2-627K mutant in virus-infected mouse organs. Proportion of amino acid 627 of PB2 of the virus in (**a**) fox brain homogenates, (**a**) the allantoic fluid of embryonated chicken eggs inoculated with fox brain homogenates, (**a**) culture medium of MDCK cells inoculated with allantoic fluid, and (**b**) the lungs and brains of mice infected with Fox/Hok/1/22 on days 3 and 6 post-infection. The light blue on the left side of the bar shows the percentage of viruses with glutamic acid (E), and the purple on the right side shows the percentage of viruses with lysine (K). Results for the eight virus-infected mice that were analyzed are shown.

H5N1 HPAIV with lysine (K) at amino acid 627 of PB2 significantly increases replication and virulence in mice, whereas glutamic acid (E) at this position is generally less virulent in mice (12). We next analyzed the proportion of viral PB2-627E/K in the lungs and brains of Fox/Hok/1/22-infected mice using RNA-seq (Fig. 4). Viruses carrying PB2-627E were dominant (i.e., >99%) in the original homogenates of dead fox brain, in the allantoic fluid of embryonated chicken eggs inoculated with fox brain homogenates, and in the culture medium of MDCK cells propagated from the allantoic fluid (Fig. 4a). In one (no. 1) of four mice at 3 dpi, PB2-627K was dominant at 81.91% in the lungs and 100% in brain, and in the 6-dpi group, one (no. 6) of the four mice had virus in which PB2-627K was dominant (83.84% and 99.88% in the lungs and brain, respectively) (Fig. 4b). However, three (nos. 2, 3, and 4) of the four mice had virus in which PB2-627E was dominant at 94.08%, 99.62%, and 96.59% in the lungs and more than 99.90% in the brain, although PB2-627K was also present in the lungs as a minor population. Furthermore, three (nos. 5, 7, and 8) of the four mice had virus in which PB2-627E was dominant at 97.96%, 86.70%, and 86.41% in the lungs and more than 99.90% in the brain (Fig. 4b). Overall, these results demonstrate that Fox/Hok/1/22 is highly virulent in mice despite lacking the PB2-627K substitution in most of the virus clones in the infected mice.

## DISCUSSION

Clade 2.3.4.4b H5N1 HPAIV has caused tremendous damage worldwide, especially in poultry and wild birds, as well as many cases of infection in mammals, including carnivores, such as red foxes, in Asia, Europe, and the USA (1). It has been reported that foxes can be infected with H5N1 HPAIV by feeding on HPAIV-infected birds (14). Previously, Hiono et al. isolated clade 2.3.4.4b H5N1 HPAIV (Fox/Hok/1/22) from a dead Ezo red fox in Hokkaido, Japan (10). This was the first clade 2.3.4.4b H5N1 HPAIV isolation from a mammal in Japan. Phylogenetic similarity between virus isolated from the fox and that from birds suggests that infection can be caused by fox predation on virus-infected birds (15). Here, we investigated the biological properties of Fox/Hok/1/22 in a mammalian model. We found that the MLD_50_ of Fox/Hok/1/22 was 10^0.5^ PFU, and that Fox/Hok/1/22, like other H5N1 HPAIVs, grew in multiple organs, including the brain and respiratory tract of the virus-infected mice (Table 1). These results indicate that Fox/Hok/1/22 efficiently replicates and is highly virulent in the mouse model.

Previous studies have identified several amino acid substitutions in PB2 that play an important role in the adaptation of avian influenza viruses to mammals (13). It has recently been reported that many strains of clade 2.3.4.4b H5N1 HPAIV isolated from mammals possess the PB2-I292V, Q591K, E627K, and/or D701N amino acid substitutions, which increase viral polymerase activity and pathogenicity in mammals (1, 16, 17); however, Fox/Hok/1/22 does not have such mammalian-adapting amino acid substitutions in its PB2. Nevertheless, Fox/Hok/1/22 grew efficiently and was highly virulent in mice (Table 1; Fig. 2). Therefore, we conducted sequencing analysis to determine the frequency of variants with these amino acid substitutions in PB2 in Fox/Hok/1/22-infected mice. At 3 and 6 dpi, in one of four mice infected with Fox/Hok/1/22, the PB2-627K mutant became dominant, whereas PB2-627E-bearing virus remained dominant in the other animals (Table 2; Fig. 4b). Mice (nos. 1 and 6) with dominant PB2-627K-bearing virus appeared to have higher viral titers in their organs than mice with dominant PB2-627E-bearing virus (Table 2). PB2-I292V, Q591K, and D701N were undetectable by Sanger sequencing (Table 2), and D701N was present at very low levels in a few animals, indicating minimal relevance (data not shown). A virus with PB2-S489P was dominant in one mouse, but the function of PB2-S489P is not consistent; it may or may not increase polymerase activity in mammals depending on the subtype, and its effects may be enhanced if present in combination with NP mutations (18, 19). Taken together, these data indicate that Fox/Hok/1/22 is highly virulent to mice without acquiring the previously well-known mammalian-adapting amino acid substitutions of PB2. However, organ titers in mice in which the PB2-627K mutant appeared were higher than those in other mice, suggesting that viruses with PB2-627K replicate more efficiently in mice than those with PB2-627E. In addition, Fox/Hok/1/22 has several known virulence markers, such as PB1-F2 N66S and NP-N319K in mammals. The PB1-F2 N66S substitution has been linked to enhanced pathogenicity in mice via inhibition of interferon production (20, 21). In the H7 subtype of HPAIVs, NP-319K has been reported to be involved in pathogenicity by enhancing the efficiency of the nuclear transport of RNP by importin-alpha (22). Which amino acid substitutions play an important role in the high virulence of Fox/Hok/1/22 in mammals will be investigated in future studies.

To assess whether the observed virulence of Fox/Hok/1/22 was specific to its mammalian host, we tested a genetically similar crow-derived virus, A/crow/Hokkaido/0103B065/2022 (H5N1; Crow/Hok/B065/22), isolated from the same region and season (15). Both Fox/Hok/1/22 and Crow/Hok/B065/22 exhibited high virulence in mice with similar phenotypic characteristics (Fig. 2; Fig. S1). These findings suggest that the high virulence of the fox-derived virus cannot be solely attributed to its isolation from a mammalian host. In addition, during the 2021–2022 winter–spring season, multiple genotypes of clade 2.3.4.4b H5N1 viruses were reported in China, and some of these genotypes differ in their virulence in mice (23). Although high virulence in mice is not a universal feature of clade 2.3.4.4b viruses, the identification of strains capable of causing severe disease highlights the need to further investigate genotype- and strain-specific determinants of virulence.

Although mice are not natural hosts of avian influenza viruses and do not fully reflect receptor specificity or transmissibility in humans, the mouse model remains a widely accepted and practical system for initial *in vivo* pathogenicity assessments (24–27). We acknowledge its limitations in extrapolating to human infection, particularly with respect to transmission dynamics. Future studies using more physiologically relevant models, such as ferrets, will be needed to further evaluate the zoonotic and transmission potential of this strain.

Clade 2.3.4.4b H5N1 HPAIVs have evolved and are reported to be more diverse. To date, clade 2.3.4.4b H5N1 HPAIVs belonging to the G2b, G2c, and G2d subgroups have been isolated in Japan (28). Here, we examined the virulence of Fox/Hok/1/22, which is a G2d virus, in mice, and future studies should be performed to compare its virulence with that of viruses belonging to other subgroups. Previous studies have reported that these viruses exhibit different pathogenicity in mice and chickens (23, 29, 30). It has also been reported that clade 2.3.4.4b H5N1 HPAIVs can diversify through genetic reassortment and become more virulent in ferrets (31). Thus, there has been diversification of virus genes and variation in virus pathogenicity. Although efficient transmission among humans has not yet occurred, further outbreaks in various wild mammals should be carefully monitored, and proactive measures should be taken to prevent and treat infections with these viruses.

## Data Availability

The raw sequencing data generated in this study have been deposited in the DDBJ Sequence Read Archive (DRA) under Bioproject: PRJDB37943.

## SUPPLEMENTAL MATERIAL

Supplemental material spectrum.01097-25-s0001.docx Supplemental methods; Fig. S1.

Table S1 spectrum.01097-25-s0002.pdf Molecular markers associated with the adaptation of avian influenza viruses to mammalian hosts found in the viral proteins of Fox/Hok/1/22.

ASM does not own the copyrights to Supplemental Material that may be linked to, or accessed through, an article. The authors have granted ASM a non-exclusive, world-wide license to publish the Supplemental Material files. Please contact the corresponding author directly for reuse.
