# Supplementary material for: Characterization of H5N1 high pathogenicity avian influenza virus belonging to clade 2.3.4.4b isolated from Ezo red fox in Japan in a mouse model
Source: Microbiol Spectr. 2025 Nov 26;14(1):e01097-25. doi: 10.1128/spectrum.01097-25 (PMC12772316; doi:10.1128/spectrum.01097-25)
Supplement: Supplemental material — Supplemental methods; Fig. S1. [file spectrum.01097-25-s0001.docx]

**Supplementary Information**

**Characterization of H5N1 high pathogenicity avian influenza virus belonging to clade 2.3.4.4b isolated from Ezo red fox in Japan in a mouse model**

Shintaro Shichinohe^1^, Takahiro Hiono^2,3,4^, Yasushi Itoh^5^, Kosuke Takada^1^, Yurie Kida^1^, Pei Wang^1^, Daisuke Motooka^6^, Norikazu Isoda^2,3,4^, Ayato Takada^3,4,7^, Yoshihiro Sakoda^2,3,4,8^, and Tokiko Watanabe^1,9,10^#

**Supplementary Materials and Methods**

**Cells and Viruses**

Madin-Darby canine kidney (MDCK) cells were kindly provided by Prof. Yoshihiro Kawaoka. MDCK cells were cultured in Eagle’s minimal essential medium (MEM) containing 5% newborn calf serum (NCS). A549 (human lung epithelial) cells were obtained from American Type Culture Collection (ATCC) and maintained in Ham’s F-12K medium (Wako, Osaka, Japan) containing 10% fetal calf serum (FCS) at 37℃ in 5% CO_2_. DF-1 cells were obtained from ATCC and maintained in DMEM medium (Nacalai tesque, Kyoto, Japan) containing 10% FCS at 39℃ in 5% CO_2_. A/Crow/Hokkaido/0103B065/2022 (Crow/Hok/B065/22; H5N1) (1) was isolated in 10-day-old chicken eggs from dead crow and then propagated for mouse infection and titrated by use of plaque assays on MDCK cells cultured in Eagle’s MEM containing 0.3% BSA and 1 μg/ml *N*-*p*-tosyl-L-phenylalanin chloromethyl ketone (TPCK)-treated trypsin. Virus stocks were stored at –80℃ until use. All viral experiments were performed under biosafety level 3 (BSL3) conditions and approved by the Institutional Review Board of the Research Institute for Microbial Diseases, The University of Osaka (protocol number: BIKEN-00311-005).

**Animal experiments**

Five-week-old female BALB/c mice were obtained from Japan SLC (Shizuoka, Japan). To determine the 50% mouse lethal dose (MLD_50_), the mice were intranasally inoculated with 0.1, 1, 10, 10^2^, or 10^3^ plaque-forming units (PFU) (in 50 μl) of Crow/Hok/B065/22 under isoflurane anesthesia. Body weight change and survival were monitored daily for 14 days. Virus-infected mice were euthanized if they lost more than 25% of their initial body weight. MLD_50_ values were calculated according to the method of Reed and Muench. All animal experiments were approved by the Animal Research Committee of the Research Institute for Microbial Diseases, The University of Osaka (approval number, R04-04-0).

**Supplementary Figure**


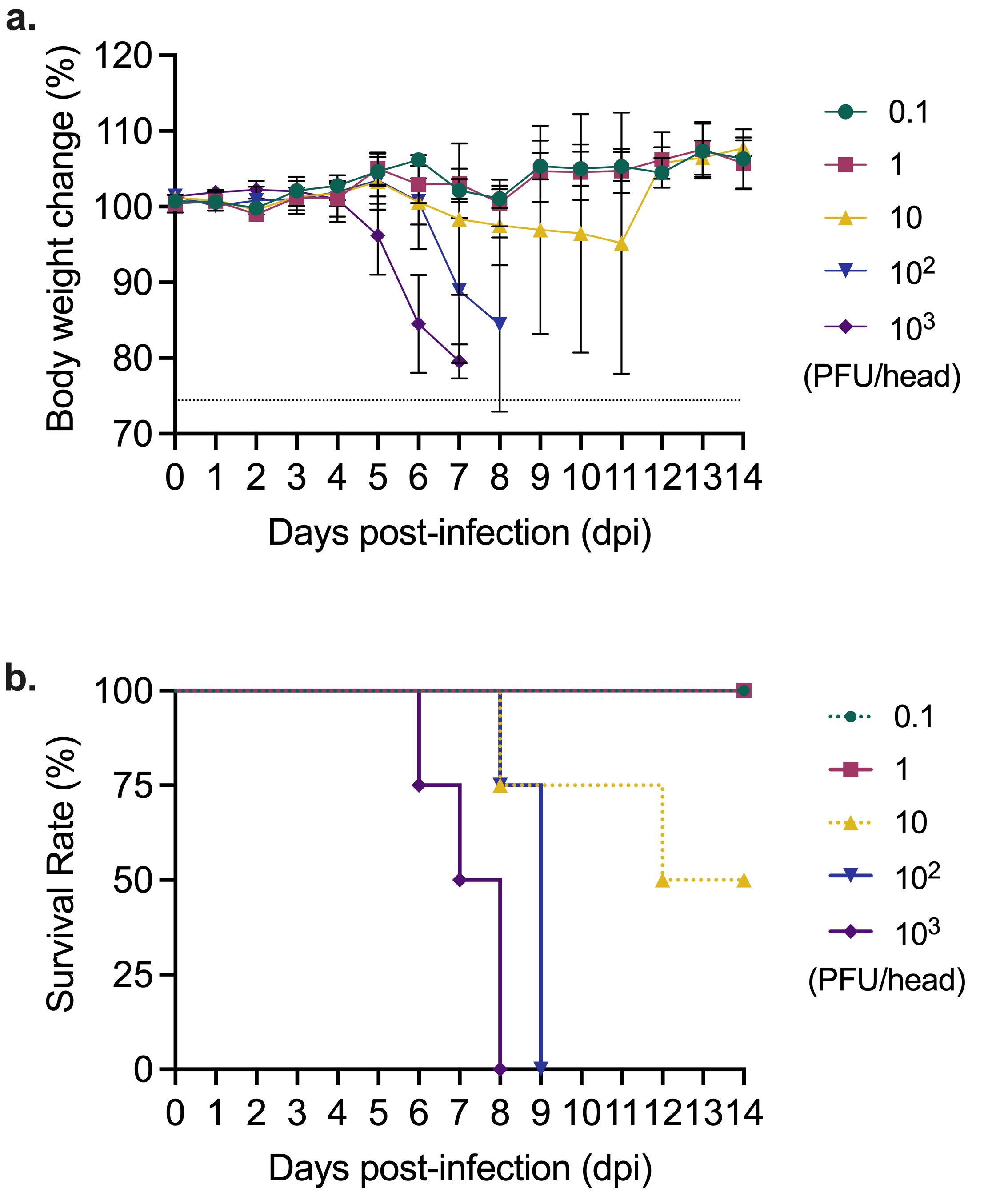


**Figure S1. Pathogenicity of Crow/Hok/B065/22 in mice.**

Mice were infected with 0.1 to 10^3^ PFU (in 50 μl) of Crow/Hok/B065/22. (a) Body weight changes were monitored daily from -2 to 14 days post-infection (dpi). The average weight of the four mice in each group from -2 dpi to 0 dpi was calculated as 100%. Body weight changes are shown as the mean ± s.d. of 4 mice per group. (b) Survival was calculated as death if the animal died or lost more than 25% of its initial body weight and had to be euthanized.

**Supplementary References**

1. Isoda N, Onuma M, Hiono T, Sobolev I, Lim HY, Nabeshima K, Honjyo H, Yokoyama M, Shestopalov A, Sakoda Y. 2022. Detection of New H5N1 High Pathogenicity Avian Influenza Viruses in Winter 2021-2022 in the Far East, Which Are Genetically Close to Those in Europe. Viruses 14:2168.
