# Supplementary material for: Characterization of H5N1 high pathogenicity avian influenza virus belonging to clade 2.3.4.4b isolated from Ezo red fox in Japan in a mouse model
Source: Microbiol Spectr. 2025 Nov 26;14(1):e01097-25. doi: 10.1128/spectrum.01097-25 (PMC12772316; doi:10.1128/spectrum.01097-25)
Supplement: Table S1 — Molecular markers associated with the adaptation of avian influenza viruses to mammalian hosts found in the viral proteins of Fox/Hok/1/22. [file spectrum.01097-25-s0002.pdf]

Table S1: Molecular markers associated with the adaptation of avian influenza viruses to mammalian hosts found in the viral proteins of Fox/Hok/1/22.

Protein	Amino acid			Viruses tested in this study	Phenotype	Subtypes tested	References
	Residue	Avian-like motif	Mammalian-like motif	Fox/Hok/1/22			
PB2	9	D	N	D	Increased virulence in mice	H5N1	Craef KM et al., 2010, Kim JH et al., 2010
	25	V	A	V	Increased virulence in mice	H5N1	Zhao D et al., 2015
	147	M	L	I	Increased virulence in mice	H9	Wang et al., 2012
	158	E	G	E	Increased polymerase activity in mammalian cell line, increased virulence in mice	H5N2, H5N9	Zhou B et al., 2011
		E	K		Increased polymerase activity and replication in mammalian and avian cell lines, increased virulence in mice	H4N6	Xu Z et al., 2019
	192	E	K	E	Increased polymerase activity in mammalian and avian cell lines, increased virulence in mice	H5N1	Taft AS et al., 2015
	199	A	S	A	Increased virulence in mice	H5N1	Kim JH et al., 2010
	253	D	N	D	Increased polymerase activity in mammalian cell line	H9N2	Zhang J et al., 2018
	256	D	G	D	Enhanced polymerase activity, mammalian host adaptation	H5N1	Manzoor et al., 2009
	271	T	A	T	Increased polymerase activity in mammalian and avian cell lines, increased virulence in mice	H3N2(avian)	Bussey KA et al., 2010
						H5N1	Foeglein A et al., 2011
						H7N9	Mok CKP et al., 2014
	292	I	V	I	Increased polymerase activity in mammalian and avian cell lines, increased virulence in mice	H9N2	Gao W et al., 2019
					Increased polymerase activity in mammalian cell line	H10N8	Xiao C et al., 2016
	389	K	R	K	Increased polymerase activity and replication in mammalian cell line	H7N9	Hu M et al., 2017
	404	F	L	F	Increased virulence in mice	H9	Liu et al., 2014
	482	K	R	R	Increased polymerase activity in mammalian cell line	H7N9	Yamayoshi et al., 2014, Yamayoshi et al., 2018
	504	I	V	V	Increased virulence in mice	H9	Kandeik et al., 2014
	526	K	R	K	Increased polymerase activity in mammalian cell line	H5N1, H7N9	Song et al., 2014
	535	M	L	M	Increased polymerase activity in mammalian cell line	H7N9	Chen G-W et al., 2016
	588	A	V	A	Increased polymerase activity and replication in mammalian and avian cell lines, increased virulence in mice	H7N9,H9N2.H10N8	Xiao C et al., 2016
	591	Q	K	Q	Enhanced replication efficiency and increased virulence in mice	H5N1	Yamada et al., 2010
					Increased polymerase activity and replication in mammalian cell line, increased virulence in mice	H9N2	284
	598	V	T/I	T	Increased polymerase activity and replication in mammalian cells, increased virulence in mice	H7N9	Hu M et al., 2017
	627	E	K	E	Increased replication efficiency in cell culture and enhanced virulence in mice	H5N1	Hatta et al., 2007
					Increased virulence in mice	H5N1	Kim et al., 2010
					Increased virulence in mice	H5N1	Chen et al., 2007
					Enhanced polymerase activity and mammalian host adaptation	H5N1	Manzoor et al., 2009
					Increased virulence in mice	H5N1	Hatta et al., 2001; Shinya et al., 2004
					Increased virulence in mice	H5N1	Fornek et al., 2009
					Mammalian host adaptation	H5N1	Le et al., 2009
					Mammalian host adaptation, increased virulence in mice	H5N1	Mase et al., 2006
					Enhanced polymerase activity	H5N1	Bortz et al., 2011
					Mammalian host adaptation	H5N1	Bogs et al., 2011
					H5 virus transmissible among ferrets	H5N1	Herfst et al., 2012
					Increased polymerase activity and replication in mammalian cells, increased virulence in mice	H4N6	Xu G et al., 2019
					Increased polymerase activity and in mammalian cells, increased virulence in mice	H6N1	Cheng K et al., 2014
					Increased polymerase activity and in mammalian cells, increased virulence in mice	H7N7	Sederi H et al., 2016, de Jong RM et al., 2013
					Increased polymerase activity and replication in mammalian cells, increased virulence in mice	H7N9	Sederi H et al., 2016, Zhang et al., 2014
					Increased polymerase activity and in mammalian cells, increased virulence in mice	H9N2	Sederi H et al., 2016, Sang X et al., 2015
		E	V		Increased polymerase activity and replication in mammalian cells, increased virulence in mice	H5N1	Taft AS et al., 2015
	701	D	N	D	Increased viral replication in mammalian cells and virulence in mice	H1N2	Yu Z et al., 2019
					Enhanced replication efficiency and transmission in guinea pigs	H5N1	Gao et al., 2009
					Mammalian host adaptation	H5N1	Le et al., 2009
					Enhanced replication efficiency; increased virulence and transmission in guinea pigs	H5N1	Steel et al., 2009
					Mammalian host adaptation, increased virulence in mice	H5N1	Li et al., 2005
					Increased polymerase activity in mammalian cell line	H7N9	Chen G-W et al., 2016, Mok CKP et al., 2014
					Increased polymerase activity in mammalian cell line	H9N2	Sederi H et al., 2016,
		D	V	D	Increased polymerase activity and replication in mammalian cells, increased virulence in mice	H5N1	Taft AS et al., 2015
	714	S	R	S	Increased polymerase activity and replication in mammalian cells	H7N7	Sederi H et al., 2016, Gabriel G et al., 2005, Gabriel G et al., 2007
					Increased polymerase activity in mammalian cells	H9N2	Sederi H et al., 2016

	89, 309	89	L	V	V	Increased polymerase activity and replication in mammalian cells, increased virulence in mice	H5N1	Li et al., 2009
		309	G	D	D			
	89, 309, 339,477, 495,627,676,	89	L	V	V	Enhanced polymerase activity and increased virulence in mice	H5N1	Li et al., 2009
		309	G	D	D			
		339	T	K	K			
		477	R	G	G			
		495	I	V	V			
		627	K	E	E			
		676	A	T	T			
	147, 627	147	M	L	I	Increased polymerase activity and increased virulence in mice	H9N2	Wang J et al., 2012
		627	E	K	E			
	147, 339, 588	147	I	T	I	Increased polymerase activity and increased virulence in mice	H5N1	Fan S et al., 2014
		339	K	T	K			
		588	A	T	A			
	340,588	340	R	K	R	Transmission in guinea pigs	H9N2	Liu et al., 2019
		588	A	V	A			
	368, 391,447,627	368	R	Q	R	Reduction in replication efficiency in cell culture and virulence in mice and ferrets	H5N1	Salomon et al., 2006
		391	Q	E	E			
		447	Q	H	Q			
		627	K	Q	E			
	526, 627	526	K	R	K	Increased polymerase activity and viral replication in mammalian cell lines, increased virulence in mice	H5N1, H7N9	Song W et al., 2014
		627	E	K	E			
	627, 701	627	E	K	E	Increased polymerase activity in mammalian cell line	H7N9	Zhu W et al., 2015
		701	D	N	D			
	627, 714	627	E	K	E	Increased polymerase activity in mammalian cell lines	H7N7, H7N9, H9N2	Sederi H et al., 2016
		714	S	R	S			
	627, 701, 714	627	E	K	E	Increased polymerase activity in mammalian cell line, increased virulence in mice	H9N2	Cuzudai-Matwich V et al., 2014
		701	D	N	D			
		714	S	R	S			
	701, 714	701	D	N	D	Increased polymerase activity in mammalian cell line, increased virulence in mice	H5N1,H7N7, H7N9, H9N2	Sederi H et al., 2016
		714	S	R	S			
	627 (with HA: H110Y, T160A, Q226L, G228S; PB1:H99Y)		E	K	E	Enable airborne transmissibility between ferrets and contact transmission between guinea pigs	H5N1	Herfst et al., 2012, Linster M et al., 2014
PB1	3		D	V	V	Increased polymerase activity and viral replication in avian and mammalian cell lines	H5N1	Elgendt EM et al., 2017
	105		N	S	N	Increased polymerase activity and viral replication in mammalian cell lines, increased virulence in mice	H5N1	Taft AS et al., 2015
	577		K	E	K	Increased polymerase activity and increased virulence in mice	H9N2	Kamiki H et al., 2018
	622		D	G	G	Increased polymerase activity and increased virulence in mice	H5N1	Feng X et al., 2016
	677		T	M	T	Increased polymerase activity in mammalian cells, decrease replication efficiency, decreased virulence in mice	H5N1	Li J et al., 2011
	678		S	N	S	Increased polymerase activity and viral replication in mammalian cell lines	H7N7	Gabriel et al., 2007
	99, 368	99	H	Y	H	H5 virus transmissible among ferrets	H5N1	Herfst et al., 2012
		368	I	V	I			
	99 (with HA:110Y, T160A, G226L, G228S, PB2:E627K)		H	Y	H	Airborne transmissible in ferrets	H5N1	Herfst et al., 2012, Linster M et al., 2014
	37		S	A	A	Increased polymerase activity in mammalian cell line	H7N9	Yamayoshi et al., 2014
	63		V	I	V	Increased polymerase activity and enhanced replication in mammalian cell line, increased virulence in mice	H7N7	Hu M et al., 2016, Hu M et al.,2017
	97		T	I	T	Increased polymerase activity and replication in mammalian cell line, increased virulence in mice	H5N1	Taft AS et al., 2015
							H5N2	Song M-S et al., 2009
							H6N1	Cheng K et al., 2014
	142		K	N/E	K	Increased virulence in mice	H5N1	Kim JH et al., 2010
	158		K	R	K	Increased polymerase activity in mammalian cell line	H5N1	Elgendt EM et al., 2017
	356		K	R	K	Increased polymerase activity and replication in mammalian cell line, increased virulence in mice	H9N2	Xu G et al., 2019
	383		N	D	D	Increased polymerase activity in avian and mammalian cell lines	H5N1	Song J et al., 2011, Song J et al., 2015

PA	409		N	S	S	Increased polymerase activity and replication in mammalian cell line	H7N9	Yamayoshi et al., 2014
	421		S	I	S	Increased virulence in mice	H5N1	Kim JH et al., 2010
	443		R	K	R	Increased virulence in mice	H5N1	Zhao D et al., 2015
	497		K	R	K	Increased polymerase activity in mammalian cell line	H7N9 (human isolate)	Yamayoshi et al., 2018
	615		K	N	K	Increased polymerase activity and increased virulence in mice	H7N7	Gabriel G et al., 2005, Gabriel G et al., 2007
	34, 347	34	A	S	K	Increased polymerase activity and increased virulence in mice	H5N1	Zhou G et al., 2018
		347	D	E	D			
	142, 147, 171, 182	142	K	R	K	Increased polymerase activity in mammalian cell line	H7N9	Liang L et al., 2019
		147	I	V	I			
		171	I	V	I			
		182	M	L	M			
	44, 127,241, 343, 573	44	V	I	V	Increased polymerase activity and increased virulence in mice	H5N1	Yamaji et al., 2015
		127	V	A	V			
		241	C	Y	C			
		343	A	T	A			
		573	I	V	I			
	149, 226, 357, 515	149	S	P	S	Increased polymerase activity in mammalian cell line	H5N1	Leung BW et l., 2010
		226	H	R	L			
		357	I	K	T			
		515	S	T	T			
	356 (with PB2 E627K)		K	R	K (PB2-627E)	Increased polymerase activity, enhanced replication in mammalian cell line, increased virulence in mice	H9N2	Xu G et al., 2016
HA (a)	101		D	N	S	Increased virus binding to α2-6	H5N1	Su et al., 2008
	126		S	N	S	Increased virus binding to α2-6	H5N1	Wang et al., 2010
	143		G	R	G	Increased virus binding to α2-6	H5N1	Yamada et al., 2006
	158		S	N	N	Increased virus binding to α2-6	H5N1	Wang et al., 2010
	159		S	N	D	Increased virus binding to α2-6	H5N1	Wang et al., 2010
	160		T	A	A	Increased virus binding to α2-6, increased transmission ability in guinea pigs	H5N1	Wang et al., 2010; Wang et al.,2015
	176		N	S	L	Increased virulence in mice	H9	Park et al, 2015
	186		V	N	N	Increased virus binding to α2-6, decrease binding to α2-3	H13N6	Lu et al., 2013
	187		D	G	N	Increased virus binding to α2-6	H5N1	Chen et al., 2012
	189		T	A	E	Enhanced replication in ferrets, transmitted via aerosols among ferrets	H9	Sorrel et al., 2009
	190		T	V	E	Enhanced binding affinity to mammalian cells and replication in mammalian cells	H9N2	Teng et al., 2016
	192		R	G	T	Enhanced replication in ferrets, aerosols transmission among ferrets	H9	Sorrel et al., 2009
	193		K	S	N	Swine, mice; increased virus binding to α2-6, enhanced replication in mammalian cells	H9	Yang et al., 2017
			K	R/T		Increased virus binding to α2-6	H5N1	Wang et al 2010; Peng et al., 2018
	196		Q	R/H	K	Increased virus binding to α2-6	H5N1	Chen et al.,2017,204,214
	197		N	K	N	Increased virus binding to α2-6	H5N1	Yamada et al., 2006
	198		N	T	P	Increase replication and transmission in ferrets	H9	Kaverin et al., 2004
	200		T	A	T	Swine; increased virus binding to α2-6	H9	Peiris et al.,1997; Lloren et al., 2017
	212		T	I	R	Swine cell lines, mice; increased virus binding to α2-6	H9	Yang et al., 2017
	214		V	I	V	Increased virus binding to α2-6	H5N1	Watanabe et al., 2011
	225		G	D	G	Increased virus binding to α2-6	H6N1	De Vries et al., 2017
	226		Q	L	Q	Increased virus binding to α2-6, enhanced replication in mammalian cells and ferrets, enhanced contact transmission in ferrets	H9N2	Wan H et al., 2007, Wan H et al., 2008
	227		Q	P	R	Increasing virus binding to α2-6, increased tropism in Guinea pigs, increased replication in mammalian cell lines	H9	Sang et al., 2015
	230		P	S	M	Increased virus binding to α2-6	H5N1	Watanabe et al., 2011
	387		K	I	K	Decreased pH of fusion, increased HA stability, increased replication efficiency and virulence in mice	H5N1	Abdelwhab et al.,2016; Reed et al., 2009, Krenn et al., 2011
	HA-2: 202		R	G	I	Enhanced airborne transmission in ferrets	H9	Sorrel et al., 2009
	189, 192	189	T	A	E	Enhanced replication in ferrets, transmitted via aerosol among ferrets	H9N2 (with human H3N2 backbone)	Sorrel et al., 2009
		192	G	R	T			
	41		I	V	I	Increased polymerase activity in mammalian cell line	H7N9	Zhu W et al., 2015
	210		E	D	E			
	227		K	R	K	Increased polymerase activity in mammalian cell line	H5N1	Chen L et al., 2017
	229		K	R	K			
	319		N	K	K	Enhanced replication efficiency in mammalian cell	H5N1	Gabriel et al., 2008
	357 (with PB2 627K)		Q	K	Q	Enhanced virulence in mice	H5N1	Kim et al., 2010

NP	470		K	R	K	Increased polymerase activity, replication in mammalian cell line, increased virulence in mice	H5N1	Chen L et al., 2017
	99,345	99	N	K	R	Increased virulence in mice	H5N1	Herfst et al., 2012
		345	S	N	S			
	434 (with HA: Q227P, D375E)		E	K	E	Enhanced transmission in guinea pigs	H9N2	Sang X et al., 2015
	434 (with HA: Q227P, PB2: D195N)							
	99, 345 (with HA: H110Y, T160A, Q226L, G228S; PB2:E627K; PB1 H99Y, I368V)	99	R	K	R	Airborne transmissible in ferrets	H5N1	Herfst S et al., 2012
		345	S	N	S			
NA	49-73 (stalk region)		No deletion	Δ (b)	No deletion	Enhanced virulence in mice	H5N1(H1N1 back bone), H7N9 (human isolated)	Matsuoka et al., 2009; Zhou et al.,2009; Bi et al., 2016
						Enhanced virulence in mice	H5N1, H7N9 (human isolated)	Li et al.,2011; Bi et al., 2016,Park et al., 2017
M1	30		N	D	D	Increased virulence in mice	H5N1	Fan et al., 2009
	43		I	M	M	Increased virulence in mice, chickens, and ducks	H5N1	Nao N et al., 2015
	139		Y	A	T	Increased virulence in mice	H5N1	Smeenk, 1996; Brown, 1999
	215		T	A	A	Increased virulence in mice	H5N1	Fan et al., 2009
NS1	2		D	N	D	Increase replication and transmission in ferrets	H9	Kimble et al., 2014
	42		P	S	S	Increased virulence in mice	H5N1	Jiao et al., 2008
	80-83	80	T	Δ	T	Increased virulence in mice	H5N1	Long et al., 2008; Seo et al., 2002
		81	I		I			
		82	A		A			
		83	S		P			
	87		D	E	S	Increased virulence in mice	H5N1	Long et al., 2008; Seo et al., 2002
	98		L	F	M	Increased virulence in mice	H5N1	Kuo and Krug 2009; Spesock et al., 2011
	101		I	M	D	Increased virulence in mice	H5N1	Kuo and Krug 2009; Spesock et al., 2011
	138		C	F	F	Increased replication mammalian cells, decreased interferon response	H5N1	Li J et al., 2018
	149		V	A	A	Increased virulence and decreased interferon response	H5N1	Li Z et al., 2009
	103, 106	103	L	F	F	Increased virulence in mice	H5N1	KuoR-L et al., 2009
		106	I	M	M			
	55, 66, 138	55	K	E	E	Enhanced replication in mammalian cells, decreased Interferon response	H5N1	Li J et al., 2018
		66	K	E	E			
		138	C	F	F			
	227-230	227	E	R	E	Increased virulence in mice	H5N1	Jackson et al., 2008
		228	S	S	S			
		229	E	E/K	E			
		230	V	V	V			
		227	E	R	E	Increased viral replication in human and duck cell lines but no effect in murine cells	H7N1	Soubies SM et al., 2010
		228	S	S	S			
		229	E	K	E			
		230	V	V	V			
NEP/NS2	16		M	I	M	Increased polymerase activity in mammalian cell line	H5N1	Reuther P et al., 2014

(a) HA residue positions are presented with H3 HA numbering.

(b) (Δ) indicates deletion of the amino acid at that position.
